# Relationship between sympathoadrenal and pituitary-adrenal response during colorectal distention in the presence of corticotropin-releasing hormone in patients with irritable bowel syndrome and healthy controls

**DOI:** 10.1371/journal.pone.0199698

**Published:** 2018-07-06

**Authors:** Yukari Tanaka, Motoyori Kanazawa, Michiko Kano, Manabu Tashiro, Shin Fukudo

**Affiliations:** 1 Department of Behavioral Medicine, Tohoku University Graduate School of Medicine, Sendai, Japan; 2 Department of Frontier Research Institute for Interdisciplinary Sciences, Tohoku University Graduate School of Medicine, Sendai, Japan; 3 Cyclotron RI Center, Tohoku University Graduate School of Medicine, Sendai, Japan; University of California Los Angeles, UNITED STATES

## Abstract

Corticotropin-releasing hormone (CRH) mediates stress responses in the brain-gut axis. Administration of CRH modulates brain activation, for example by controlling the autonomic nervous system in response to colorectal distention. Here, we investigated the relationship between sympathoadrenal and hypothalamic-pituitary-adrenal (HPA) responses to colorectal distention in patients with irritable bowel syndrome (IBS). We enrolled 32 patients with IBS (16 women and 16 men) and 32 healthy subjects (16 women and 16 men), and randomly divided them between CRH and saline injection groups. The patients randomly underwent no (0 mmHg), mild (20 mmHg), or strong (40 mmHg) colorectal distension. CRH (2 μg/kg) or saline was then administered via injection, and the distention protocol was repeated. The heart rate (HR) and HR variability (HRV; calculated as the low [LF] to high frequency [HF] peak ratio, LF/HF) were analyzed using electrocardiography. Plasma noradrenaline, adrenaline, adrenocorticotropic hormone (ACTH), and cortisol levels were measured at the time of each distention. Plasma adrenaline levels were shown to be associated with plasma ACTH levels in HCs injected with CRH during distention using structural equation modeling analysis. Patients with IBS injected with placebo during distention displayed a closer association between these two parameters than those injected with CRH. Generalized estimating equation analysis revealed a significant distention × group × drug interaction for HF power. Moreover, there was a strong correlation between adrenaline and HRV upon CRH injection in controls, but not patients with IBS. The relationship between HPA-sympathoadrenal responses and CRH levels during colorectal distention differs between patients with IBS and controls. Modulation of adrenal gland activity in response to ACTH stimulation may contribute to the brain-gut pathophysiology characteristic of IBS.

## Introduction

Irritable bowel syndrome (IBS) is a functional gastrointestinal disorder characterized by abdominal pain and altered bowel function in the absence of structural or inflammatory abnormalities [1, 2]. Its prevalence is high, and affects approximately 15% of adults [1, 2]. As the abdominal symptoms in IBS are closely linked to psychological stress, the combined functioning of intestinal motor, sensory, and central nervous activities is thought to be an important factor in the development of IBS symptoms [3]. Corticotropin-releasing hormone (CRH) is a major mediator of the stress response in the brain-gut axis, and it increases colonic motility and sensitivity [4–6]. Stress results in CRH release in the paraventricular nucleus of the hypothalamus, as well as secretion of adrenocorticotropic hormone (ACTH) from the pituitary, which stimulates the adrenal gland to release cortisol [7]. Patients with IBS have excess levels of released ACTH in plasma and cortisol in serum in response to stress [4, 8], although suppressed basal levels of plasma ACTH have also been reported in patients with IBS [9]. Increased hypothalamic-pituitary-adrenal (HPA) responses to stress alter the balance between afferent and efferent neural pathways. We have found that activity in brain regions implicated in CRH secretion is suppressed during strong colorectal distention in men with IBS [10]. Sex differences in central nervous system responses to visceral stress, perception, and motility have been reported in patients with IBS. Enhanced cortisol and ACTH responses to CRH have been observed in men with IBS when compared with healthy controls (HCs), while women have been reported to have blunted responses [11]. It has also been reported that male patients with IBS likely exhibit a ceiling effect with regard to their responses to colorectal distention both in the amygdala and in plasma noradrenaline levels following CRH injection [10].

These findings seem to agree with parallel modifications in the HPA and catecholamine pathways [7]. In addition, the catalytic activity of phenylethanolamine N-methyltransferase (PNMT), which is the enzyme that synthesizes adrenaline from noradrenaline, is regulated by cortisol, and stress induces adrenal PNMT activity [12]. Experiments using nociceptive stressors have revealed a strong correlation between ACTH levels and catecholamine levels [13]. Nevertheless, how the HPA-catecholamine network is modulated during stress in patients with IBS remains unknown. The noradrenaline system hub is found in the locus coeruleus (LC), which sends neural projections to the amygdala and hypothalamus, which in turn contain numerous CRH receptors. The LC also sends major projections to the frontal cortex, which is able to regulate the amygdala [14]. Differences in CRH responses to regional brain activation, especially in the amygdala (which combines visceral sensation and negative emotion) [15], have been reported between patients with IBS and HCs [10, 16]. CRH is an important cause of noradrenergic release during stress, especially in the hypothalamus, LC, and amygdala [17–19]. These brain regions modulate responses to emotional arousal and regulate the autonomic nervous system. Heart rate variability (HRV) is a measure obtained noninvasively using electrocardiography, and is used to assess autonomic nervous activity [20, 21]. HRV analysis during colonic distention has revealed decreased vagal and increased sympathetic nerve activity in patients with IBS when compared with HCs [21]. In fact, the vagal component of the HRV is negatively correlated with the motility index in patients with IBS [22]; therefore, ANS activity is a good indicator for brain-gut interactions.

The purpose of the present study was to determine the influence of CRH on stress-neuroendocrine networks, such as the HPA axis and sympathoadrenal network. We hypothesized that (1) plasma noradrenaline and adrenaline levels were more strongly associated with plasma ACTH levels in response to CRH injection during colorectal distention; and (2) plasma catecholamine and ACTH levels were positively correlated with HRV, and CRH further strengthened this relationship in patients with IBS when compared with controls.

## Materials and methods

### Participants

Thirty-two patients with IBS (16 women and 16 men; age: mean ± standard deviation [SD], 21.7 ± 1.6 years) and 32 sex- and age-matched HCs (16 women and 16 men; age: mean ± SD, 22.0 ± 2.1 years) participated in this study. All male subjects, except for two subjects with IBS that were administered the CRH injection, had participated in our earlier study [10]. Electrocardiography data from two subjects were not correctly recorded; therefore, these two subjects were excluded, and two new age- and IBS subtype-matched male patients were recruited to the CRH administration group. The neuroendocrine data from the men presented in this study are almost identical to those presented in our previous study, while the HRV data have not yet been presented. The hypothesis in this study and methods used for data analysis here are very different our previous study [10]. Here, we present a network comparison between HRV and neuroendocrine levels. We also present combined data from men and women. This led to higher statistical power in our analysis.

All of the patients with IBS were diagnosed based on the Rome lll criteria [1]. The IBS group included 22 patients (9 women and 13 men) with diarrhea, 3 (2 women and 1 man) with constipation, and 7 (5 women and 2 men) with mixed IBS. Each participant was screened for evidence of other gastrointestinal diseases, autonomic or cardiovascular diseases, mental disorders, and traumatic history. The HCs were free from gastrointestinal symptoms. None of the participants had any organic diseases or mental disorders. No drugs were prescribed to the participants, and they reported not taking any medication for one week before the study. Written informed consent was obtained from all participants prior to their participation. This study was approved by the Ethics Committee of the Tohoku University Graduate School of Medicine, Japan.

The State-Trait Anxiety Inventory [23] and Self-Rating Depression Scale [24] were administrated to all participants before the day of the experiment to assess their anxiety and depression levels. There were no significant differences in state anxiety (IBS, 41.3 ± 8.6 vs. HC, 39.0 ± 13.4; t (30) = .57; *P* = .50; Cohen’s d = .26; 95% confidence interval [CI], -10.4 to 5.9), trait anxiety (IBS, 46.1 ± 9.9 vs. HC, 43.3 ± 11.3; t (30) = .77; *P* = .48; Cohen’s d = .18; 95% CI, –10.5 to 4.8), or self-rating depression scale scores (IBS, 36.3 ± 5.7 vs. HC, 35.7 ± 6.4; t (30) = .26; *P* = .14; Cohen’s d = .10; 95% CI, –4.9 to 3.8) between the female IBS group and the female HC group.

### Distention protocol

We used the barostat protocol as previously described [10, 25, 26]. A bag was positioned 10 cm from the anal verge of the subjects, and its distal end was inserted into the colorectum. The catheter was then connected to computerized barostat equipment (Synectics Visceral Stimulator; Medtronics Synectics; Shoreview, MN). Intravenous catheters were inserted into each side of the cubital vein and the barostat bag was inserted into the colorectum of each subject 30 min before the study. The experimental session consisted of two stages: stage 1, without injection of CRH or saline; and stage 2, following injection of CRH (Tanabe-Mitsubishi; Osaka, Japan) or saline. The protocol was administered for 80 s under the following conditions: baseline (0 mmHg), no distention (0 mmHg), mild (20 mmHg) colorectal distention, or strong (40 mmHg) colorectal distention. The interval between the two stimuli was 15 min due to radiotracer decay. During each stage, the baseline condition was assessed first, and was followed by one of the other three conditions in a random order. After stage 1, half of the subjects in each group (IBS or HC) were administered either human CRH (2 μg/kg, dissolved in 2 ml saline) or saline alone as a bolus. The same distention protocol was then repeated. The subjects were informed that the different conditions were selected in a random order, and were blinded to the timing and the group that they were assigned to. The CRH dose used has previously been shown to alter gastrointestinal motility and increase plasma ACTH secretion, but has not been associated with any side effects [10]. Blood samples were obtained from an intravenous cannula after each period, and subjective symptoms were evaluated using an ordinate scale [10]. The plasma and serum were centrifuged for 5 min at 1,500 *g*, and then frozen and stored at –30°C for later analysis.

### HR and HRV

Holter electrocardiography data were continuously sampled (SCM 6000; Fukuda Denshi; Tokyo, Japan), and colorectal distention was recorded using a specific key input. R-R intervals during the distention were calculated using computer software (R-R Interval Analyzing Program, HPS-RRA; Fukuda Denshi). HRV intervals were calculated based on frequency domain analysis for 64 s during colorectal distention. Low frequency bands (LF, 0.04–0.15 Hz), high frequency bands (HF, 0.15–0.4 Hz), and the LF/HF ratio were analyzed to assess sympathetic and parasympathetic activity. HF reflects the parasympathetic tone, while the LF/HF ratio reflects the sympathetic/parasympathetic balance [19, 27, 28].

### Neuroendocrine data

Plasma ACTH and serum cortisol levels were determined by radioimmunoassay, and plasma noradrenaline and adrenaline levels were measured using high-performance liquid chromatography, as previously reported [4, 5, 26].

### Statistical analysis

The sample size in this study was calculated based on the sample size determination theory of statistics. Sample size was calculated using an effect size of 0.75, beta value of 0.2, and alpha value of 0.05. Demographic data were analyzed using SPSS 21.0 (IBM Corporation; Armonk, NY, USA). All data are presented as mean ± SD. The significance level threshold was set to *P* < 0.05. The data were analyzed using the non-parametric Student’s t-test and the Spearman rank correlation coefficient to account for non-normal distributions of some variables. An overall generalized estimating equation (GEE) analysis [29] (SPSS 21.0, IBM Corp.) was performed during the random distention. The fixed main effects included those of group (IBS or HC), drug (CRH or saline), condition (no distention, 20 mmHg distention, or 40 mmHg distention), and sex (female or male), as well as their potential interactions with the dependent variables of interest. Network analyses within the neuroendocrine system were conducted using structural equation modeling in Amos 22.0 (IBM Corp.). The strengths of relationships between two factors are indicated as standardized beta weights using an arrow path. A satisfactory model usually has a comparative fit index ≥ 0.95 and a root mean square error of approximation < 0.05. The significance level threshold was set to *P* < .0125.

## Results

### Neuroendocrine networks of the HPA and adrenal axes

All hormonal responses in the female participants are shown in the **S1 Text, S1 and S2 Fig, S1 Table** and the data from the male participants has been reported previously [10]. We found sex-based differences in plasma noradrenaline levels, but not in plasma ACTH, serum cortisol, or plasma adrenaline. As a result, the following analyses were performed on data from each administration subgroup in the IBS and HC groups, including both men and women. Consistent patterns emerged in the correlational analyses between plasma ACTH, serum cortisol, plasma noradrenaline, and adrenaline levels **(S2 Table)**. In HCs receiving a placebo injection, plasma ACTH and serum cortisol levels remained positively correlated during no distention (rho = .576, *P* = .019) and 20 mmHg distention (rho = .548, *P* = .028) conditions. Plasma ACTH and noradrenaline levels were significantly negatively correlated (rho = –.609, *P* = .012) during 40 mmHg distention, but were unrelated to plasma adrenaline levels. In HCs receiving a CRH injection, ACTH levels were significantly correlated with adrenaline levels in the no distention condition (rho = .583, *P* = .018). ACTH levels in patients with IBS receiving a placebo injection were significantly and positively correlated with cortisol levels (no distention: rho = .624, *P* = .010; 20 mmHg distention: rho = .716, *P* = .002; 40 mmHg distention: rho = .818, *P* < .001). ACTH levels were also significantly correlated with noradrenaline levels during 20 mmHg distention (rho = .556, *P* = .025), and adrenaline levels during 40 mmHg distention (rho = .605, *P* = .013). Administration of CRH in patients with IBS did not affect the ACTH-cortisol or ACTH-adrenaline relationships in 0 and 20 mmHg distention conditions. ACTH and plasma adrenaline levels were not significantly correlated in these participants in the 40-mmHg distention condition.

We observed a significant group × drug × condition × sex interaction for plasma noradrenaline levels **(S1 Text, S1 Table)**; therefore, we analyzed the correlation between plasma noradrenaline and other neuroendocrine variables in each sex group. There was a significant correlation between plasma adrenaline and noradrenaline during 40 mmHg distention in male HCs receiving a CRH injection (rho = .881, *P* = .004). However, there were no significant correlations with plasma noradrenaline in female participants during any of the random distension conditions.

### Associations between neuroendocrine networks assessed using structural equation modeling

**Fig 1:**
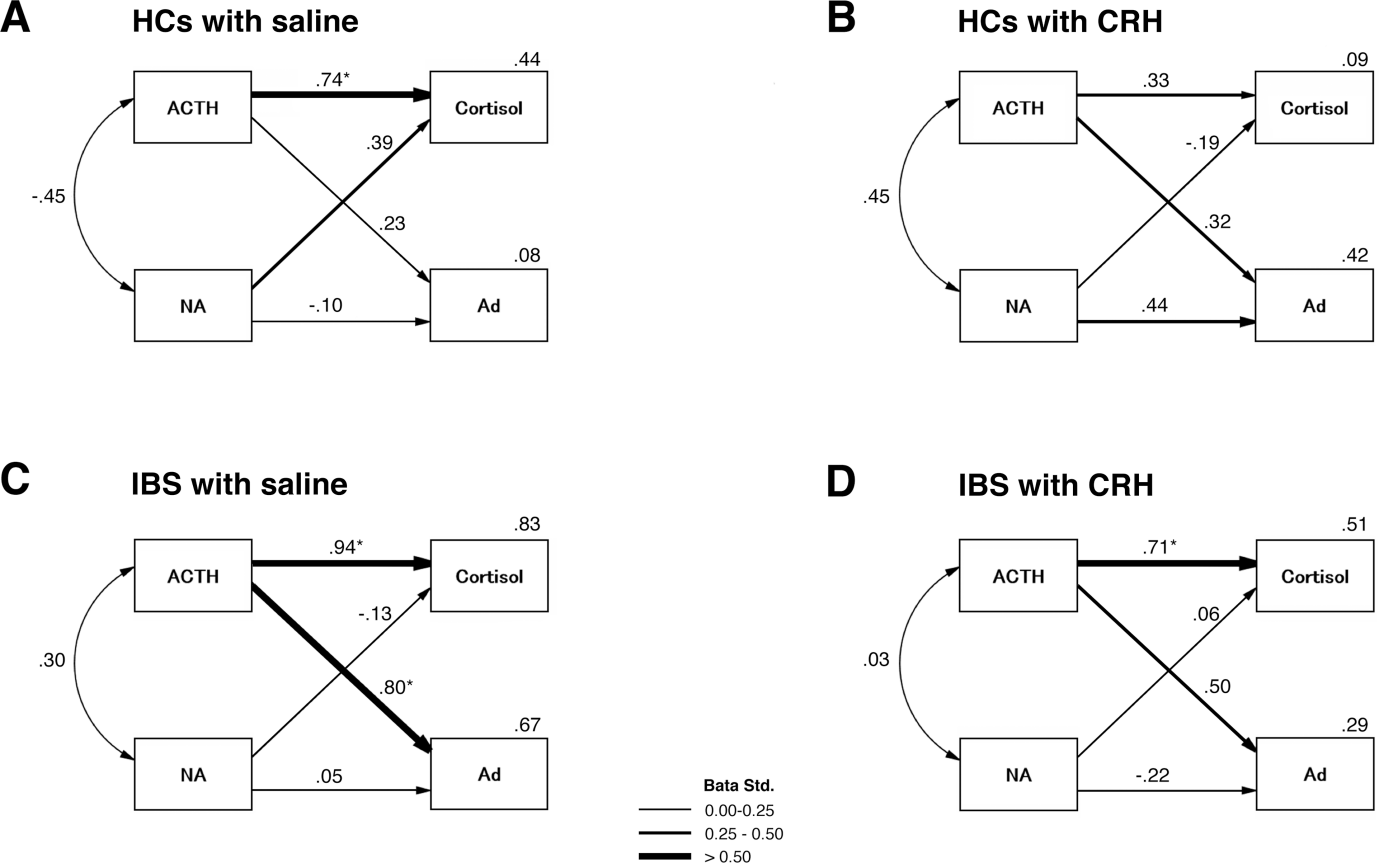
Neuroendocrine response models during 40 mmHg distention. (**A**) HCs injected with saline (n = 16), (**B**) HCs injected with CRH (n = 16), (**C**) patients with IBS injected with saline (n = 16), and (**D**) patients with IBS injected with CRH (n = 16). *P < .0125 indicate significant paths. The squared multiple correlations (R^2^) of the variables are reported in the top right corner. There were no significant factor correlations between ACTH and NA. ACTH, plasma ACTH; cortisol, serum cortisol; HCs, healthy controls; NA, plasma noradrenaline; Ad, plasma adrenaline; ACTH, adrenocorticotropic hormone; IBS, irritable bowel syndrome; CRH, corticotropin-releasing hormone.

Structural equation modeling was used to better understand the relationship between ACTH-cortisol and noradrenaline-adrenaline, assess network differences between IBS and HC groups, and examine the effect of CRH on these relationships. This model included standardized coefficients for each relationship in the analysis. Each coefficient is presented over the respective pathway represented by the arrows in **Fig 1**. The model fit was tested together for the CRH and saline groups in both the IBS and HC groups (χ^2^(4) = 1.706, *P* = .790, comparative fix index = 1.000, root mean square error of approximation = .000, 95% CI = 0.000–0.126). As expected, higher plasma ACTH levels in patients with IBS were associated with higher levels of serum cortisol (β = .94, *P* < .001); we found a similar association with plasma adrenaline levels (β = .972, P < .001) during strong distention in patients with IBS who were administered saline, and plasma ACTH levels in patients with IBS patients who were administered CRH (cortisol: β = .711, *P* < .001; adrenaline: β = .496, *P* < .001). In contrast, in HCs, plasma ACTH levels were significantly associated with serum cortisol levels in the placebo group (β = .744, *P* < .001). In addition, plasma noradrenaline and adrenaline levels were significantly associated in the CRH injection group (β = .439, *P* = .046). Structural equation modeling during no distention and 20 mmHg distention showed a significant association between plasma ACTH and serum cortisol, in addition to plasma adrenaline in IBS with or without CRH (**S3 and S4 Fig**).

### HRV

**Fig 2:**
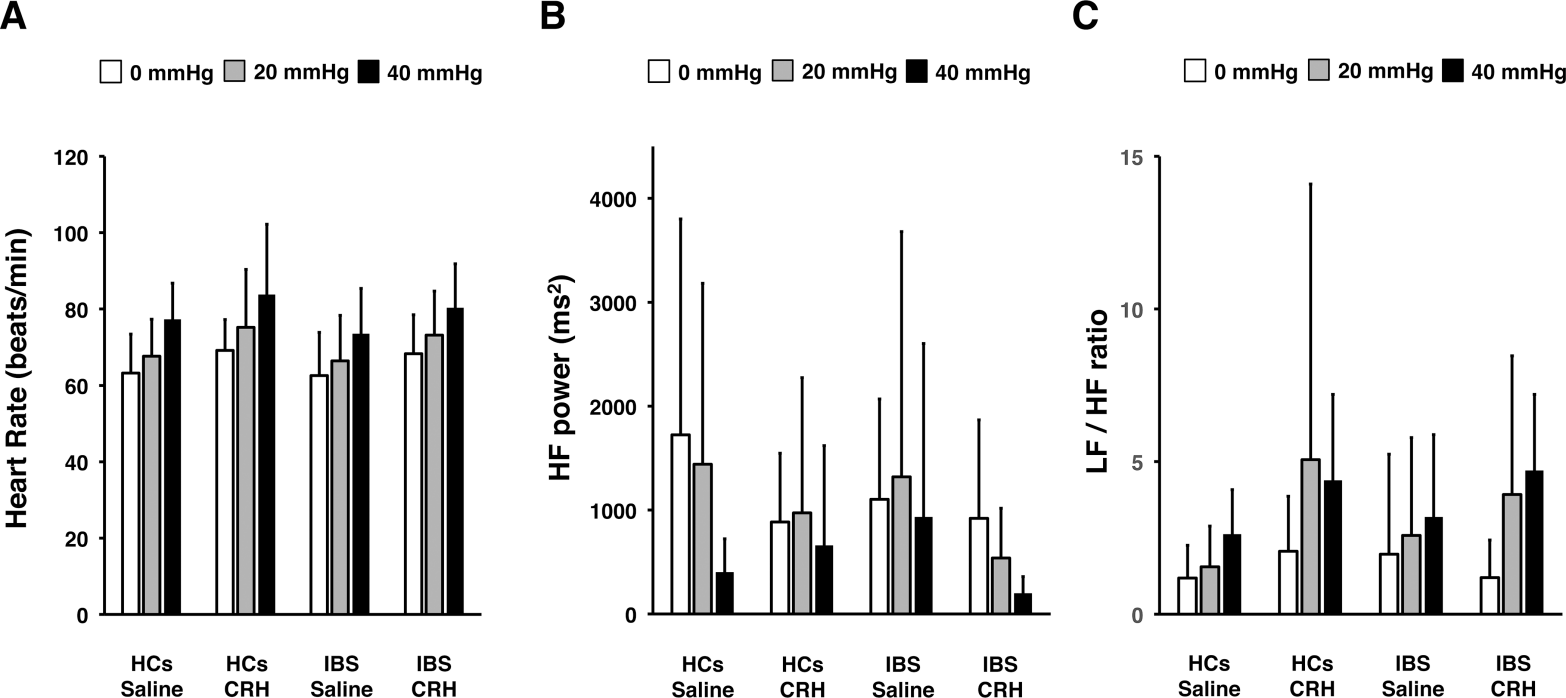
Effects of CRH on HRV during random distention after injection. (**A**) HR (beats/min), (**B**) HF power, and (**C**) LF/HF ratio in HCs administered with saline (n = 16) or CRH (n = 16); and patients with IBS administered with saline (n = 16) or CRH (n = 16). Results are represented as mean ± SD. GEE analysis of HRV parameters during random distention revealed a significant distention × group × drug interaction for HF power (*P* = .016). LF, low frequency; HF, high frequency; HCs, healthy controls.

**Fig 3:**
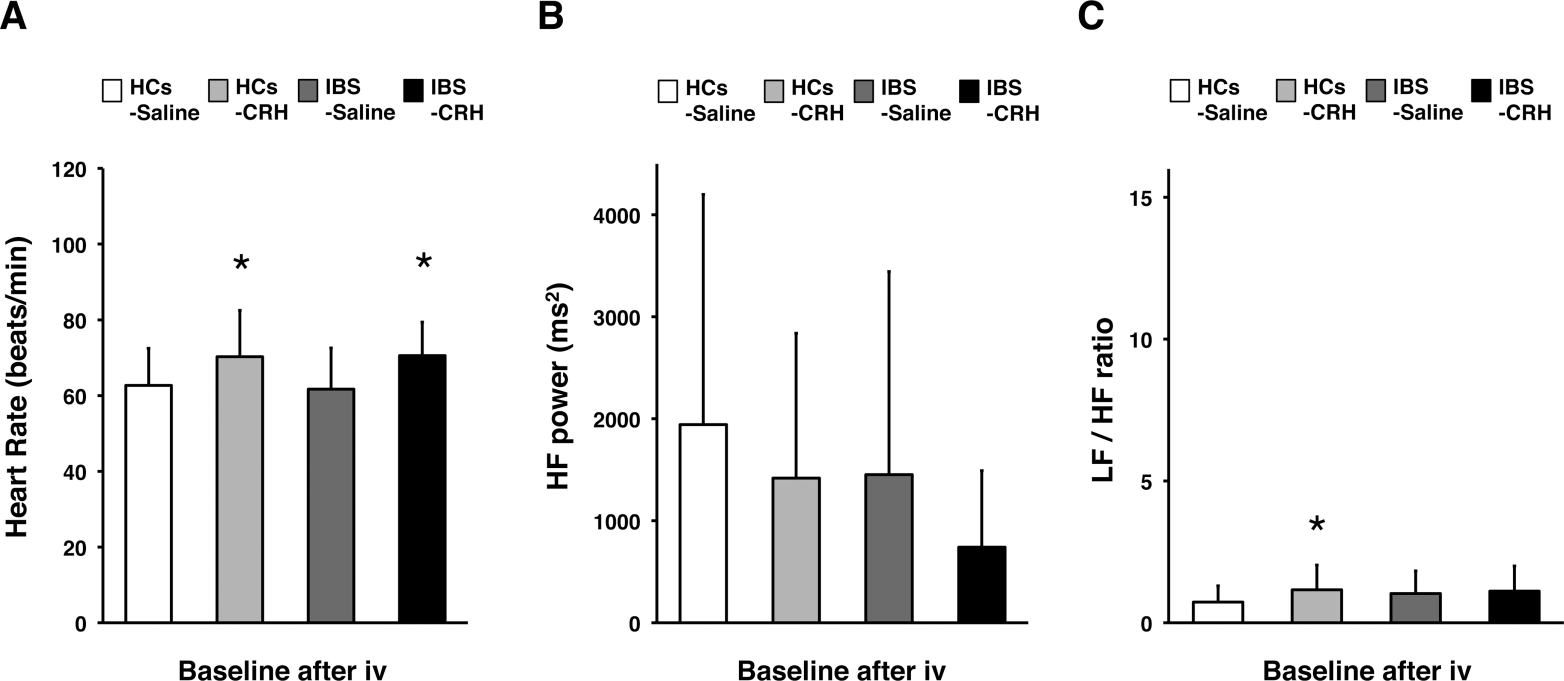
Effects of CRH on HRV during the baseline period after injection. (**A**) HR (beats/min), (**B**) HF power, and (**C**) LF/HF ratio in HCs administered with saline (n = 16) or CRH (n = 16); and patients with IBS administered with saline (n = 16) or CRH (n = 16). Results are represented as mean ± SD. *P < .05 when compared with placebo; baseline after CRH or saline injection, paired t-test. LF, low frequency; HF, high frequency; HCs, healthy controls.

To explore the effects of CRH on autonomic nervous responses to colonic stimuli, we performed a GEE analysis (**Fig 2**). There was a significant distention × group × drug interaction (*P* = .016) for HF power, but not HR or LF/HF ratio (HR, *P* = .939; LF/HF ratio, *P* = .408). There were no significant distention × group × drug × sex interactions with any of the HRV parameters (HR, *P* = .295; HF, *P* = .197; LF/HF ratio, *P* = .110). Basal HR, HF power, and the LF/HF ratio were not significantly different between the IBS and HC groups (**Fig 3**). HR was significantly higher in HCs receiving a CRH injection than in HCs treated with placebo (CRH, 70.3 ± 11.2 beats/min; placebo, 61.5 ± 9.0 beats/min; *P* = .021), and in patients with IBS receiving a CRH injection when compared with those treated with placebo (CRH, 70.5 ± 9.2 beats/min; placebo, 61.7 ± 12.6 beats/min; *P* = .031). The LF/HF ratio was also higher in controls receiving a CRH injection than in those receiving a placebo injection (CRH, 1.2 ± 0.8; placebo, 0.6 ± 0.5; *P* = .040).

### Correlation between the neuroendocrine stress response and HRV

We used Spearman’s correlation analysis to investigate the relationship between CRH-influenced pituitary-cortisol or catecholamine responses and HRV during stress (**Table 1**). We found no correlation between neuroendocrine responses and HRV in HCs receiving a placebo injection. However, there were significant correlations between plasma adrenaline levels and HR, as well as HF power, for all levels of distention, in HCs who were administered CRH. In addition, there was a significant correlation between the LF/HF ratio and plasma adrenaline levels in these participants in the 40-mmHg distention condition. The plasma adrenaline levels and HRV were not correlated in patients with IBS. There was a significant correlation between HR and plasma ACTH levels, as well as serum cortisol levels, in the 40-mmHg distention condition in the IBS placebo group.

**Table 1:** Features of HRV during each distention and correlation between HRV and neuroendocrine variables.

Variable		Mean	SD	ACTH		Cortisol		Ad		NA	
**A. During no distention**											
HC with placebo injection (n = 16)											
	HR	62.7	10.19	-0.05		-0.42		0.39		-0.24	
	HF power	1825.53	2101.22	-0.07		0.12		-0.19		0.32	
	LF/HF	1.16	1.12	0.31		0.12		0.32		-0.32	
HC with CRH injection (n = 16)											
	HR	69.15	8.35	0.54	*	0.46		0.58	*	0.19	
	HF power	884.81	684.14	-0.71	**	-0.37		-0.68	**	-0.28	
	LF/HF	2.06	1.86	0.47		0.08		0.36		0.32	
IBS with placebo injection (n = 16)											
	HR	62.58	11.66	0.54	*	0.11		0.54	*	0.55	*
	HF power	1104.01	996.33	-0.27		-0.24		-0.41		-0.48	
	LF/HF	1.97	3.38	-0.09		-0.16		0.28		0.14	
IBS with CRH injection (n = 16)											
	HR	68.32	10.49	0.04		0.12		-0.11		-0.33	
	HF power	919.88	977.99	-0.11		-0.23		0.26		0.22	
	LF/HF	1.20	1.27	0.28		0.56	*	0.23		-0.34	
**B. During 20 mm Hg distention**											
HC with placebo injection (n = 16)											
	HR	67.09	9.99	-0.09		-0.29		0.35		-0.16	
	HF power	1550.92	1766.87	0.23		0.15		-0.01		-0.28	
	LF/HF	1.46	1.41	0.07		-0.14		-0.10		-0.14	
HC with CRH injection (n = 16)											
	HR	75.18	15.67	0.28		0.11		0.78	**	0.15	
	HF power	973.36	1343.16	0.07		-0.18		-0.69	**	-0.20	
	LF/HF	5.06	9.33	-0.34		-0.14		0.04		-0.25	
IBS with placebo injection (n = 16)											
	HR	66.45	12.31	0.62	*	0.47		0.35		0.42	
	HF power	1319.21	2438.16	-0.54	*	-0.36		-0.13		-0.44	
	LF/HF	2.58	3.31	0.27		0.06		0.22		0.34	
IBS with CRH injection (n = 16)											
	HR	73.18	11.9	0.06		0.41		0.16		-0.22	
	HF power	538.99	493.39	-0.06		-0.51	*	-0.22		-0.05	
	LF/HF	3.92	4.69	0.16		0.62	*	0.46		0.17	
**C. During 40 mm Hg distention**											
HC with placebo injection (n = 16)											
	HR	77.23	9.89	0.04		-0.28		0.45		-0.12	
	HF power	437.89	338.40	-0.11		0.21		-0.39		-0.04	
	LF/HF	2.48	1.55	-0.37		-0.02		-0.31		0.42	
HC with CRH injection (n = 16)											
	HR	83.73	19.08	0.25		0.44		0.77	**	0.37	
	HF power	658.02	993.43	-0.41		-0.51	*	-0.72	**	-0.29	
	LF/HF	4.38	2.92	0.10		0.20		0.66	**	0.38	
IBS with placebo injection (n = 16)											
	HR	73.48	12.3	0.58	*	0.54	*	0.22		0.17	
	HF power	931.64	1725.64	-0.22		-0.05		0.10		-0.28	
	LF/HF	3.18	2.79	-0.13		-0.38		-0.24		0.25	
IBS with CRH injection (n = 16)											
	HR	80.27	11.96	0.21		0.21		0.44		-0.12	
	HF power	197.78	167.7	0.18		-0.07		-0.27		0.04	
	LF/HF	4.70	2.59	0.04		0.28		0.41		0.01	

Data are shown as rho scores of the Spearman rank correlation coefficient. Data for %HF or LF/HF were used to assess the correlations with plasma ACTH, serum cortisol, plasma adrenaline, and noradrenaline levels during (A) no distention, (B) 20 mm Hg distention, and (C) 40 mm Hg distention.

*P < 0.05

**P < 0.01.

HR, heart rate; HF, high frequency; LF, low frequency; Ad, adrenaline; NA, noradrenaline; ACTH, adrenocorticotropic hormone; SD, standard deviation.

**Fig 4:**
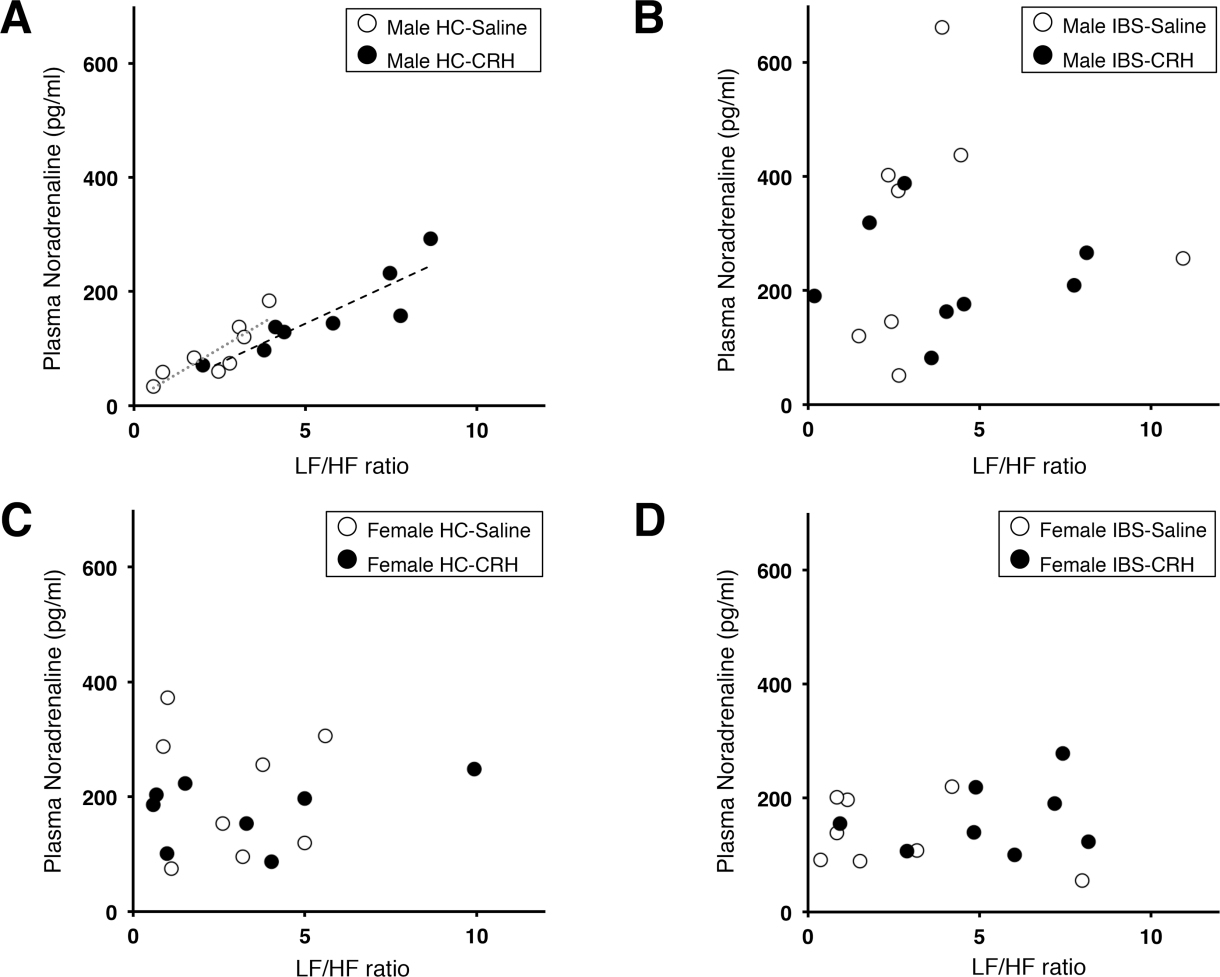
Effects of CRH on the correlation between plasma noradrenaline levels and the LF/HF ratio in men and women. Plasma noradrenaline levels and LF/HF ratio during 40 mmHg distention **(A)** in male controls injected with placebo (n = 8) and CRH (n = 8). There were significant correlations in both groups (CRH, rho = 0.95, *P* < 0.01; placebo, rho = 0.91, *P* < 0.01). (**B**) Male patients with IBS injected with placebo (n = 8) and CRH (n = 8). **(C)** Female controls injected with placebo (n = 8) and CRH (n = 8). **(D)** Female patients with IBS injected with placebo (n = 8) CRH (n = 8). No significant correlations are shown in panels **B, C,** and **D.** IBS, irritable bowel syndrome; CRH, corticotropin-releasing hormone; LF, low frequency; HF, high frequency.

**S1 Text** showed the GEE analysis of plasma noradrenaline levels revealed a significant effect of sex; therefore, we examined the correlation between plasma noradrenaline and HRV in men and women. During strong distention, plasma noradrenaline levels were significantly correlated with only the LF/HF ratio in male HCs receiving CRH (rho = 0.95, *P* < 0.01) or saline (rho = 0.91, *P* = 0.002) injections, but not in men with IBS. There were no significant correlations between HRV and neuroendocrine levels in women with IBS or female HCs (**Fig 4**).

Ordinate scales for subjective symptoms were shown in **S2 Text**.

## Discussion

In the present study, we showed an association between plasma adrenaline ACTH levels in HCs injected with CRH during strong distention. Patients with IBS injected with placebo displayed a closer association between these two parameters than those injected with CRH during strong distention. However, plasma noradrenaline levels were not significantly associated with ACTH or adrenaline levels. Plasma adrenaline levels were strongly correlated with HRV in HCs receiving CRH and patients with IBS receiving a placebo. In contrast, plasma ACTH and noradrenaline levels were not correlated with HRV. We found a modification in plasma adrenaline responses to colorectal distention, which suggested that the adrenal gland could potentially underlie the autonomic dysfunction characteristic of IBS. Our findings regarding plasma adrenaline responses to colorectal distention showed significant correlations between ACTH and HRV, especially in HCs with CRH, although there was no correlation associated with plasma noradrenaline. Injection of CRH amplifies the sympathetic output in response to colorectal distension, which appears linear in HCs. In contrast, there is an apparent ceiling effect in patients with IBS and for some markers, such as adrenaline, indicated by no additional effect of distension. These results might reflect the homeostatic response to elevated CRH in IBS.

We found that CRH injection accelerated ACTH and cortisol release in the IBS and HC groups, which is supported by the results of Dinan et al. [8], and those of our previous study [4]. Moreover, the threshold for ACTH-induced adrenaline release was enhanced only during strong distention in HCs. In contrast, there was a correlation in the IBS group, even in the absence of distention. Colonic nociceptive signaling first sends information to the dorsal root ganglia through fine sympathetic afferent fibers [30, 31]. Adrenergic stimulation mediates the induction of visceral hypersensitivity; thus, the blockage of α1/α2- and β1/β2-adrenergic receptors prevents the induction of visceral hypersensitivity [31]. Increased expression of nerve growth factor in the colon has been shown to alter the electrophysiological characteristics of dorsal root ganglion neurons through voltage-gated sodium channels [31–33]. CRH receptors in the colonic mucosa also mediate the expression of nerve growth factor in colonic tissues [31]. These neurons form synapses in the lamina of the dorsal horn of the spinal cord, which sends projections to the thalamus. The thalamus in turn projects to the insula, anterior cingulate cortex, amygdala, hippocampus, and hypothalamus. [30, 34]. Thus, excessive afferent neural input and more intense signaling in colonic neurons might exacerbate the intense abdominal pain sensation in patients with IBS who have been administered with CRH.

Another factor potentially mediating the effects of CRH administration is neuroendocrine-immune interactions. Following CRH administration, male HCs had higher amygdala activation during strong colorectal distention when compared with men with IBS [10]. Excessive secretion of CRH induced remodeling of neural networks and hormonal receptors, which has been termed as the “allostatic load” [35, 36]. Repeated episodes of stress also modulate adrenal medulla-cortex networks. The synthesis of adrenaline from noradrenaline is regulated by cortisol *via* PNMT, and stress induces adrenal PNMT activity [12]. Plasma noradrenaline and serum cortisol showed altered response patterns in response to CRH during colorectal distention, and a similar pattern was found between serum cortisol and plasma adrenaline. In addition, frequent intra-adrenal cortisol release stimulates the adrenal medulla [37]. These effects presumably underlie the strong association between ACTH and adrenaline responses in patients with IBS. Allosteric patterns of plasma adrenaline might have important implications for the etiology of IBS. Elenkov et al. reported that adrenaline is involved in immune homeostasis. Specifically, individuals in a 24-h excretion group with high adrenaline levels had significantly higher levels of interleukin (IL)-10 and significantly lower levels of IL-12 and tumor necrosis factor-α than those with low adrenaline levels [38]. Mast cells are found in close proximity to sensory neurons and affect the severity and frequency of abdominal pain in patients with IBS [39]. In addition, patients with IBS have proinflammatory cytokine abnormalities, such as an imbalance in the IL-10/IL-12 ratio in the blood [40] and increased IL-10 levels in the colonic mucosa [41]. Temporal acute stress suppresses inflammatory cytokines by elevating adrenaline levels. However, repeated chronic stress induces a ceiling effect for adrenaline release, which is also influenced by HPA signaling. This leads to disruptions to the immunological balance, and might enhance the low-grade inflammation of the gut, which is characteristic of IBS. These implications should be examined in future studies, including simultaneous measurements of hormone and cytokine levels.

We have provided evidence for sex-based differences in plasma noradrenaline levels, but not in plasma ACTH, serum cortisol, or plasma adrenaline levels. Noradrenaline mediates amygdala activity, and the performance of emotional memory encoding is different between men and women [42]. The marked sex differences in noradrenaline release and regional brain function might thus result in the sex-based differences in brain networks responsible for emotional arousal. It is worth noting that postmenopausal women have been reported to have lower noradrenaline levels and kinetics in the resting period [43]. Nevertheless, our plasma noradrenaline data analysis revealed a significant distention × group × drug interaction in the absence of sex-based effects. Therefore, there was a negligible effect of female menstruation in our study.

The noradrenergic system includes the LC of the midbrain, which sends neural projections to the amygdala and hypothalamus, areas of the brain that contain numerous CRH receptors. In fact, colorectal distention stimulates hippocampal noradrenaline release and visceral perception, and is suppressed by α-helical CRH or CRH receptor 1 antagonists [44]. We show that the strong correlation between plasma noradrenaline levels and LF/HF ratio in male HCs who were administered CRH shifts to the upper right, although the strength of the correlation remains. A similar result suggested that CRH injection increased the HR and LF/HF ratio in young adult male participants [45]. Moreover, plasma adrenaline levels after CRH injection had a strong correlation with HRV in the HC group in the present study. However, the interactions between noradrenaline/adrenaline and HRV were disrupted in the patients with IBS during the distention period. As such, repeated CRH exposure might be an important risk factor for adrenosympathetic modification, as well as for HPA dysregulation.

In contrast to the significant correlation between plasma noradrenaline levels and the LF/HF ratio in male HCs, there was no significant correlation in female HCs. One of the possible factors was the menstruation effect. Female HCs have higher LF/HF activity in the luteal phase than in the follicular phase [46]. Interpretation of the results herein must therefore account for the influence of the mensuration cycle in women. Other confounding factors that may have biased our results were that HRV power was influenced by physical training [47, 48], and we were unable to adjust for the potential effects of this factor in the present study. Second, there were limitations regarding baseline study characteristics, such as bowel habits, changes in personnel, time of year, and political/environmental stressors that are associated with different time periods. We obtained blood samples a few minutes after colorectal distention. Our previous data [10] indicate that the protocol used here, which eliminates the effects of time associated with different distention intensities, can be used to measure neuroendocrine changes properly. However, the time from baseline to the peak is thought to be different for each endocrine hormone. Therefore, multiple blood samples are desirable, although this introduces ethical concerns. Patients with IBS often have a wide range of co-morbidities, including anxiety or depression [49]. In addition, early life trauma influences HPA axis responsiveness [50]. In order to exclude the direct effects of psychological confounding factors, we recruited IBS subjects who had no anxiety or depression co-morbidities, and no traumatic history. Third, we have previously reported that CRH induces colonic motility and sensation regardless of IBS subtype. Therefore, there are unanswered questions regarding the sympathoadrenal network in each IBS subtype.

In conclusion, our results suggest that the waning of key HPA and adrenal pathways mediated by CRH may inhibit regulatory mechanisms. The correlation between adrenaline and HRV may be an important parameter predictive of progression to IBS and a therapeutic target.

## Supporting information

S1 Fig Effects of CRH on the hypothalamic-pituitary-adrenocortical axis and adrenal system during random distention after injection in female subjects. (**A**) Plasma ACTH (pg/ml), (**B**) serum cortisol (μg/ml), (**C**) plasma noradrenaline (pg/ml), and (**D**) plasma adrenaline (pg/ml) in female HCs injected with saline (n = 8) and CRH (n = 8); and female patients with IBS injected with saline (n = 8) and CRH (n = 8). GEE analysis revealed significant distention × group × drug interactions for all four endocrine measures during random distention. Results are represented as mean ± SD. ACTH, adrenocorticotropic hormone; IBS, irritable bowel syndrome; CRH, corticotropin-releasing hormone; HCs, healthy controls; SD, standard deviation.
(TIF)

S2 Fig Effects of CRH on the hypothalamic-pituitary-adrenocortical axis and adrenal system during baseline after injection in female subjects. (**A**) Plasma ACTH (pg/ml), (**B**) serum cortisol (μg/ml), (**C**) plasma noradrenaline (pg/ml), and (**D**) plasma adrenaline (pg/ml) in female HCs injected with saline (n = 8), and CRH (n = 8); and female patients with IBS injected with saline (n = 8) and CRH (n = 8). Results are represented as mean ± SD. *P < .05 and **P < .01 compared with placebo; baseline after IV injection, paired t-test ACTH, adrenocorticotropic hormone; IBS, irritable bowel syndrome; CRH, corticotropin-releasing hormone; HCs, healthy controls; SD, standard deviation; IV, intravenous injection.
(TIF)

S3 Fig Neuroendocrine response models during no distention. (**A**) HCs injected with saline (n = 16), (**B**) HCs injected with CRH (n = 16), (**C**) patients with IBS injected with saline (n = 16), and (**D**) patients with IBS injected with CRH (n = 16). *P < .0125 indicate significant paths. The squared multiple correlations (R^2^) of the variables are reported in the top right corner. There were no significant factor correlations between ACTH and NA. ACTH, plasma ACTH; cortisol, serum cortisol; NA, plasma noradrenaline; Ad, plasma adrenaline; ACTH, adrenocorticotropic hormone; IBS, irritable bowel syndrome; CRH, corticotropin-releasing hormone; HCs, healthy controls.
(TIF)

S4 Fig Neuroendocrine response models during 20 mmHg distention. (**A**) HCs injected with saline (n = 16), (**B**) HCs injected with CRH (n = 16), (**C**) patients with IBS injected with saline (n = 16), and (**D**) patients with IBS injected with CRH (n = 16). *P < .0125 indicate significant paths. The squared multiple correlations (R^2^) of the variables are reported in the top right corner. There were no significant factor correlations between ACTH and NA. ACTH, plasma ACTH; cortisol, serum cortisol; NA, plasma noradrenaline; Ad, plasma adrenaline; ACTH, adrenocorticotropic hormone; IBS, irritable bowel syndrome; CRH, corticotropin-releasing hormone; HCs, healthy controls.
(TIF)

S1 Table Factors and interaction results in GEE analysis of neuroendocrine variables. (DOCX)

S2 Table Means, standard deviations, and correlations for measurement scales in the SEM models. (DOCX)

S1 Text Neuroendocrine data in female participants. (DOCX)

S2 Text Ordinate scale and subjective symptoms in women. (DOCX)

## References

[1] Longstreth GF, Thompson WG, Chey WD, Houghton LA, Mearin F, Spiller RC. Functional bowel disorders. Gastroenterology. 2006;130:1480–1491. doi: 10.1053/j.gastro.2005.11.061 16678561 10.1053/j.gastro.2005.11.061

[2] Drossman DA. The functional gastrointestinal disorders and the Rome III process. Gastroenterology. 2006;130:1377–1390. doi: 10.1053/j.gastro.2006.03.008 16678553 10.1053/j.gastro.2006.03.008

[3] Tanaka Y, Kanazawa M, Fukudo S, Drossman DA. Biopsychosocial model of irritable bowel syndrome. Journal of neurogastroenterology and motility. 2011;17:131–139. doi: 10.5056/jnm.2011.17.2.131 21602989 10.5056/jnm.2011.17.2.131 PMC3093004

[4] Fukudo S, Nomura T, Hongo M. Impact of corticotropin-releasing hormone on gastrointestinal motility and adrenocorticotropic hormone in normal controls and patients with irritable bowel syndrome. Gut. 1998;42:845–849. 9691924 10.1136/gut.42.6.845 PMC1727153

[5] Sagami Y, Shimada Y, Tayama J, Nomura T, Satake M, Endo Y, et al Effect of a corticotropin releasing hormone receptor antagonist on colonic sensory and motor function in patients with irritable bowel syndrome. Gut. 2004;53:958–964. doi: 10.1136/gut.2003.018911 15194643 10.1136/gut.2003.018911 PMC1774093

[6] Fukudo S, Saito K, Sagami Y, Kanazawa M. Can modulating corticotropin releasing hormone receptors alter visceral sensitivity? Gut. 2006;55:146–148. doi: 10.1136/gut.2005.070888 16407379 10.1136/gut.2005.070888 PMC1856495

[7] Fukudo S. Hypothalamic-Pituitary-Adrenal Axis in Gastrointestinal Physiology Physiology of the Gastronintestinal Tract: Oxford: Academic Press; 2012 pp. 791–816.

[8] Dinan TG, Quigley EM, Ahmed SM, Scully P, O'Brien S, O'Mahony L, et al Hypothalamic-pituitary-gut axis dysregulation in irritable bowel syndrome: plasma cytokines as a potential biomarker? Gastroenterology. 2006;130:304–311. doi: 10.1053/j.gastro.2005.11.033 16472586 10.1053/j.gastro.2005.11.033

[9] Chang L, Sundaresh S, Elliott J, Anton PA, Baldi P, Licudine A, et al Dysregulation of the hypothalamic-pituitary-adrenal (HPA) axis in irritable bowel syndrome. Neurogastroenterol Motil. 2009;21:149–159. doi: 10.1111/j.1365-2982.2008.01171.x 18684212 10.1111/j.1365-2982.2008.01171.x PMC2745840

[10] Tanaka Y, Kanazawa M, Kano M, Morishita J, Hamaguchi T, Van Oudenhove L, et al Differential Activation in Amygdala and Plasma Noradrenaline during Colorectal Distention by Administration of Corticotropin-Releasing Hormone between Healthy Individuals and Patients with Irritable Bowel Syndrome. PLoS One. 2016;11:e0157347 doi: 10.1371/journal.pone.0157347 27448273 10.1371/journal.pone.0157347 PMC4957789

[11] Videlock EJ, Shih W, Adeyemo M, Mahurkar-Joshi S, Presson AP, Polytarchou C, et al The effect of sex and irritable bowel syndrome on HPA axis response and peripheral glucocorticoid receptor expression. Psychoneuroendocrinology. 2016;69:67–76. doi: 10.1016/j.psyneuen.2016.03.016 27038676 10.1016/j.psyneuen.2016.03.016 PMC4977028

[12] Wurtman RJ. Stress and the adrenocortical control of epinephrine synthesis. Metabolism. 2002;51:11–14. 12040535 10.1053/meta.2002.33185

[13] Goldstein DS, Kopin IJ. Adrenomedullary, adrenocortical, and sympathoneural responses to stressors: a meta-analysis. Endocr Regul. 2008;42:111–119. 18999898 PMC5522726

[14] Sara SJ. The locus coeruleus and noradrenergic modulation of cognition. Nat Rev Neurosci. 2009;10:211–223. doi: 10.1038/nrn2573 19190638 10.1038/nrn2573

[15] LeDoux J. The amygdala. Curr Biol. 17. England; 2007. pp. R868-874.

[16] Hubbard CS, Labus JS, Bueller J, Stains J, Suyenobu B, Dukes GE, et al Corticotropin-releasing factor receptor 1 antagonist alters regional activation and effective connectivity in an emotional-arousal circuit during expectation of abdominal pain. J Neurosci. 2011;31:12491–12500. doi: 10.1523/JNEUROSCI.1860-11.2011 21880911 10.1523/JNEUROSCI.1860-11.2011 PMC3399687

[17] Van Bockstaele EJ, Colago EE, Valentino RJ. Amygdaloid corticotropin-releasing factor targets locus coeruleus dendrites: substrate for the co-ordination of emotional and cognitive limbs of the stress response. J Neuroendocrinol. 1998;10:743–757. 9792326 10.1046/j.1365-2826.1998.00254.x

[18] McCall JG, Al-Hasani R, Siuda ER, Hong DY, Norris AJ, Ford CP, et al CRH Engagement of the Locus Coeruleus Noradrenergic System Mediates Stress-Induced Anxiety. Neuron. 2015;87:605–620. doi: 10.1016/j.neuron.2015.07.002 26212712 10.1016/j.neuron.2015.07.002 PMC4529361

[19] Tache Y, Bonaz B. Corticotropin-releasing factor receptors and stress-related alterations of gut motor function. J Clin Invest. 2007;117:33–40. doi: 10.1172/JCI30085 17200704 10.1172/JCI30085 PMC1716215

[20] Akselrod S, Gordon D, Ubel FA, Shannon DC, Berger AC, Cohen RJ. Power spectrum analysis of heart rate fluctuation: a quantitative probe of beat-to-beat cardiovascular control. Science. 1981;213:220–222. 6166045 10.1126/science.6166045

[21] Mazurak N, Seredyuk N, Sauer H, Teufel M, Enck P. Heart rate variability in the irritable bowel syndrome: a review of the literature. Neurogastroenterol Motil. 2012;24:206–216. doi: 10.1111/j.1365-2982.2011.01866.x 22256893 10.1111/j.1365-2982.2011.01866.x

[22] Tanaka Y, Kanazawa M, Palsson OS, Van Tilburg MA, Gangarosa LM, Fukudo S, et al Age-dependent Association between Increased Postprandial Colonic Motility and Autonomic Nervous System Activity in Patients with Irritable Bowel Syndrome. J Neurogastroenterol Motil; 2017;24:87–95. 10.5056/jnm16216 PMC5753907 29291610

[23] Spielberger CD, Gorsuch RL, Lushene RE. STAI Manual for the StateTrait Anxiety Inventory. Palo Alto,CA: Consulting Psychologist Press; 1970.

[24] Zung WW, Richards CB, Short MJ. Self-rating depression scale in an outpatient clinic. Further validation of the SDS. Arch Gen Psychiatry. 1965;13:508–515. 4378854 10.1001/archpsyc.1965.01730060026004

[25] Hamaguchi T, Kano M, Rikimaru H, Kanazawa M, Itoh M, Yanai K, et al Brain activity during distention of the descending colon in humans. Neurogastroenterol Motil. 2004;16:299–309. doi: 10.1111/j.1365-2982.2004.00498.x 15198652 10.1111/j.1365-2982.2004.00498.x

[26] Suzuki H, Watanabe S, Hamaguchi T, Mine H, Terui T, Kanazawa M, et al Brain activation associated with changes in heart rate, heart rate variability, and plasma catecholamines during rectal distention. Psychosom Med. 2009;71:619–626. doi: 10.1097/PSY.0b013e31819b69ca 19561165 10.1097/PSY.0b013e31819b69ca

[27] Buchman TG, Stein PK, Goldstein B. Heart rate variability in critical illness and critical care. Current opinion in critical care. 2002;8:311–315. 12386491 10.1097/00075198-200208000-00007

[28] Tillisch K, Mayer EA, Labus JS, Stains J, Chang L, Naliboff BD. Sex specific alterations in autonomic function among patients with irritable bowel syndrome. Gut. 2005;54:1396–1401. doi: 10.1136/gut.2004.058685 15923667 10.1136/gut.2004.058685 PMC1774694

[29] Zeger SL, Liang KY. Longitudinal data analysis for discrete and continuous outcomes. Biometrics. 1986;42:121–30. 3719049

[30] Fukudo S. IBS: Autonomic dysregulation in IBS. Nat Rev Gastroenterol Hepatol. 2013;10:569–571. doi: 10.1038/nrgastro.2013.166 23999321 10.1038/nrgastro.2013.166

[31] Winston JH, Xu GY, Sarna SK. Adrenergic stimulation mediates visceral hypersensitivity to colorectal distension following heterotypic chronic stress. Gastroenterology. 2010;138:294–304. doi: 10.1053/j.gastro.2009.09.054 19800336 10.1053/j.gastro.2009.09.054 PMC2813397

[32] Sangameswaran L, Delgado SG, Fish LM, Koch BD, Jakeman LB, Stewart GR, et al Structure and function of a novel voltage-gated, tetrodotoxin-resistant sodium channel specific to sensory neurons. J Biol Chem. 1996;271:5953–5956. 8626372 10.1074/jbc.271.11.5953

[33] Renganathan M, Cummins TR, Waxman SG. Contribution of Na(v)1.8 sodium channels to action potential electrogenesis in DRG neurons. J Neurophysiol. 2001;86:629–640. doi: 10.1152/jn.2001.86.2.629 11495938 10.1152/jn.2001.86.2.629

[34] Fukudo S, Kanazawa M. Gene, environment, and brain-gut interactions in irritable bowel syndrome. J Gastroenterol Hepatol. 2011;26:110–115. doi: 10.1111/j.1440-1746.2011.06631.x 21443722 10.1111/j.1440-1746.2011.06631.x

[35] McEwen BS. Sex, stress and the hippocampus: allostasis, allostatic load and the aging process. Neurobiol Aging. 2002;23:921–939. 12392796 10.1016/s0197-4580(02)00027-1

[36] McEwen BS, Gianaros PJ. Central role of the brain in stress and adaptation: links to socioeconomic status, health, and disease. Ann N Y Acad Sci. 2010;1186:190–222. doi: 10.1111/j.1749-6632.2009.05331.x 20201874 10.1111/j.1749-6632.2009.05331.x PMC2864527

[37] Zuckerman-Levin N, Tiosano D, Eisenhofer G, Bornstein S, Hochberg Z. The importance of adrenocortical glucocorticoids for adrenomedullary and physiological response to stress: a study in isolated glucocorticoid deficiency. J Clin Endocrinol Metab. 2001;86:5920–5924. doi: 10.1210/jcem.86.12.8106 11739465 10.1210/jcem.86.12.8106

[38] Elenkov IJ, Kvetnansky R, Hashiramoto A, Bakalov VK, Link AA, Zachman K, et al Low- versus high-baseline epinephrine output shapes opposite innate cytokine profiles: presence of Lewis- and Fischer-like neurohormonal immune phenotypes in humans? J Immunol. 2008;181:1737–1745. 18641310 10.4049/jimmunol.181.3.1737 PMC10066863

[39] Barbara G, Stanghellini V, De Giorgio R, Cremon C, Cottrell GS, Santini D, et al Activated mast cells in proximity to colonic nerves correlate with abdominal pain in irritable bowel syndrome. Gastroenterology. 2004;126:693–702. 14988823 10.1053/j.gastro.2003.11.055

[40] O'Mahony L, McCarthy J, Kelly P, Hurley G, Luo F, Chen K, et al Lactobacillus and bifidobacterium in irritable bowel syndrome: symptom responses and relationship to cytokine profiles. Gastroenterology. 2005;128:541–551. 15765388 10.1053/j.gastro.2004.11.050

[41] Chang L, Adeyemo M, Karagiannides I, Videlock EJ, Bowe C, Shih W, et al Serum and colonic mucosal immune markers in irritable bowel syndrome. Am J Gastroenterol. 2012;107:262–272. doi: 10.1038/ajg.2011.423 22158028 10.1038/ajg.2011.423 PMC3297737

[42] Poehlman ET, Toth MJ, Ades PA, Calles-Escandon J. Gender differences in resting metabolic rate and noradrenaline kinetics in older individuals. Eur J Clin Invest. 1997;27:23–28. 9041373 10.1046/j.1365-2362.1996.640620.x

[43] Van Stegeren AH, Goekoop R, Everaerd W, Scheltens P, Barkhof F, Kuijer JP, et al Noradrenaline mediates amygdala activation in men and women during encoding of emotional material. Neuroimage. 2005;24:898–909. doi: 10.1016/j.neuroimage.2004.09.011 15652324 10.1016/j.neuroimage.2004.09.011

[44] Saito K, Kasai T, Nagura Y, Ito H, Kanazawa M, Fukudo S. Corticotropin-releasing hormone receptor 1 antagonist blocks brain-gut activation induced by colonic distention in rats. Gastroenterology. 2005;129:1533–1543. doi: 10.1053/j.gastro.2005.07.053 16285953 10.1053/j.gastro.2005.07.053

[45] Arlt J, Jahn H, Kellner M, Strohle A, Yassouridis A, Wiedemann K. Modulation of sympathetic activity by corticotropin-releasing hormone and atrial natriuretic peptide. Neuropeptides. 2003;37:362–368. 14698679 10.1016/j.npep.2003.09.006

[46] Sato N, Miyake S, Akatsu J, Kumashiro M. Power spectral analysis of heart rate variability in healthy young women during the normal menstrual cycle. Psychosom Med. 1995;57:331–335. 7480562 10.1097/00006842-199507000-00004

[47] Goldsmith RL, Bigger JT Jr., Steinman RC, Fleiss JL. Comparison of 24-hour parasympathetic activity in endurance-trained and untrained young men. J Am Coll Cardiol. 1992;20:552–558. 1512332 10.1016/0735-1097(92)90007-a

[48] De Meersman RE. Heart rate variability and aerobic fitness. Am Heart J. 1993;125:726–731. 8438702 10.1016/0002-8703(93)90164-5

[49] Whitehead WE, Palsson O, Jones KR. Systematic review of the comorbidity of irritable bowel syndrome with other disorders: what are the causes and implications? Gastroenterology. 2002;122:1140–1156. 11910364 10.1053/gast.2002.32392

[50] Videlock EJ, Adeyemo M, Licudine A, Hirano M, Ohning G, Mayer M, et al Childhood trauma is associated with hypothalamic-pituitary-adrenal axis responsiveness in irritable bowel syndrome. Gastroenterology. 2009;137:1954–1962. doi: 10.1053/j.gastro.2009.08.058 19737564 10.1053/j.gastro.2009.08.058 PMC2789911

