# Supplementary figures and images for: Relationship between sympathoadrenal and pituitary-adrenal response during colorectal distention in the presence of corticotropin-releasing hormone in patients with irritable bowel syndrome and healthy controls
Source: PLoS One. 2018 Jul 6;13(7):e0199698. doi: 10.1371/journal.pone.0199698 (PMC6034822; doi:10.1371/journal.pone.0199698)

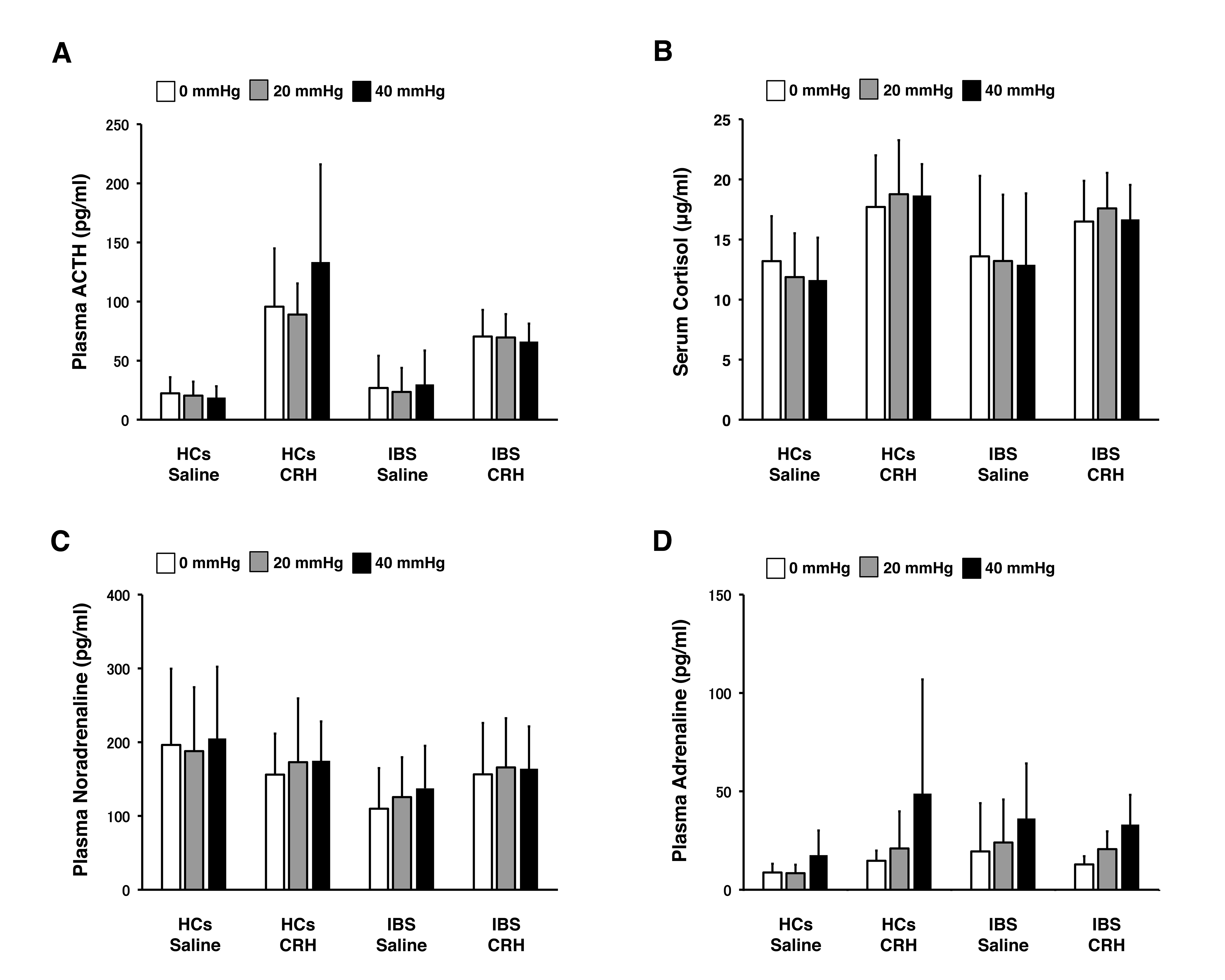

Supplement: S1 Fig — (A) Plasma ACTH (pg/ml), (B) serum cortisol (μg/ml), (C) plasma noradrenaline (pg/ml), and (D) plasma adrenaline (pg/ml) in female HCs injected with saline (n = 8) and CRH (n = 8); and female patients with IBS injected with saline (n = 8) and CRH (n = 8). GEE analysis revealed significant distention × group × drug interactions for all four endocrine measures during random distention. Results are represented as mean ± SD. ACTH, adrenocorticotropic hormone; IBS, irritable bowel syndrome; CRH, corticotropin-releasing hormone; HCs, healthy controls; SD, standard deviation. (TIF) [file pone.0199698.s001.tif]

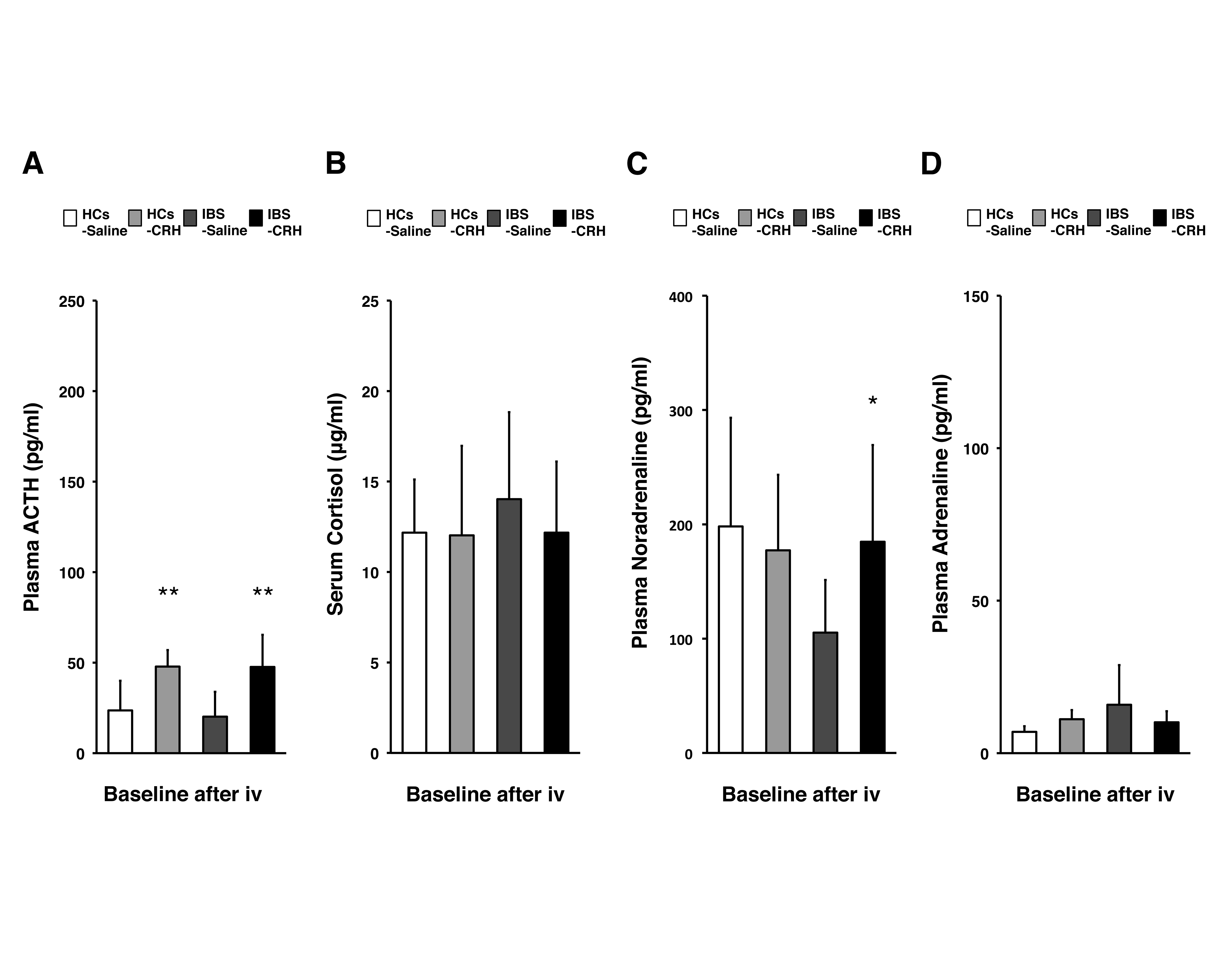

Supplement: S2 Fig — (A) Plasma ACTH (pg/ml), (B) serum cortisol (μg/ml), (C) plasma noradrenaline (pg/ml), and (D) plasma adrenaline (pg/ml) in female HCs injected with saline (n = 8), and CRH (n = 8); and female patients with IBS injected with saline (n = 8) and CRH (n = 8). Results are represented as mean ± SD. *P < .05 and **P < .01 compared with placebo; baseline after IV injection, paired t-test ACTH, adrenocorticotropic hormone; IBS, irritable bowel syndrome; CRH, corticotropin-releasing hormone; HCs, healthy controls; SD, standard deviation; IV, intravenous injection. (TIF) [file pone.0199698.s002.tif]

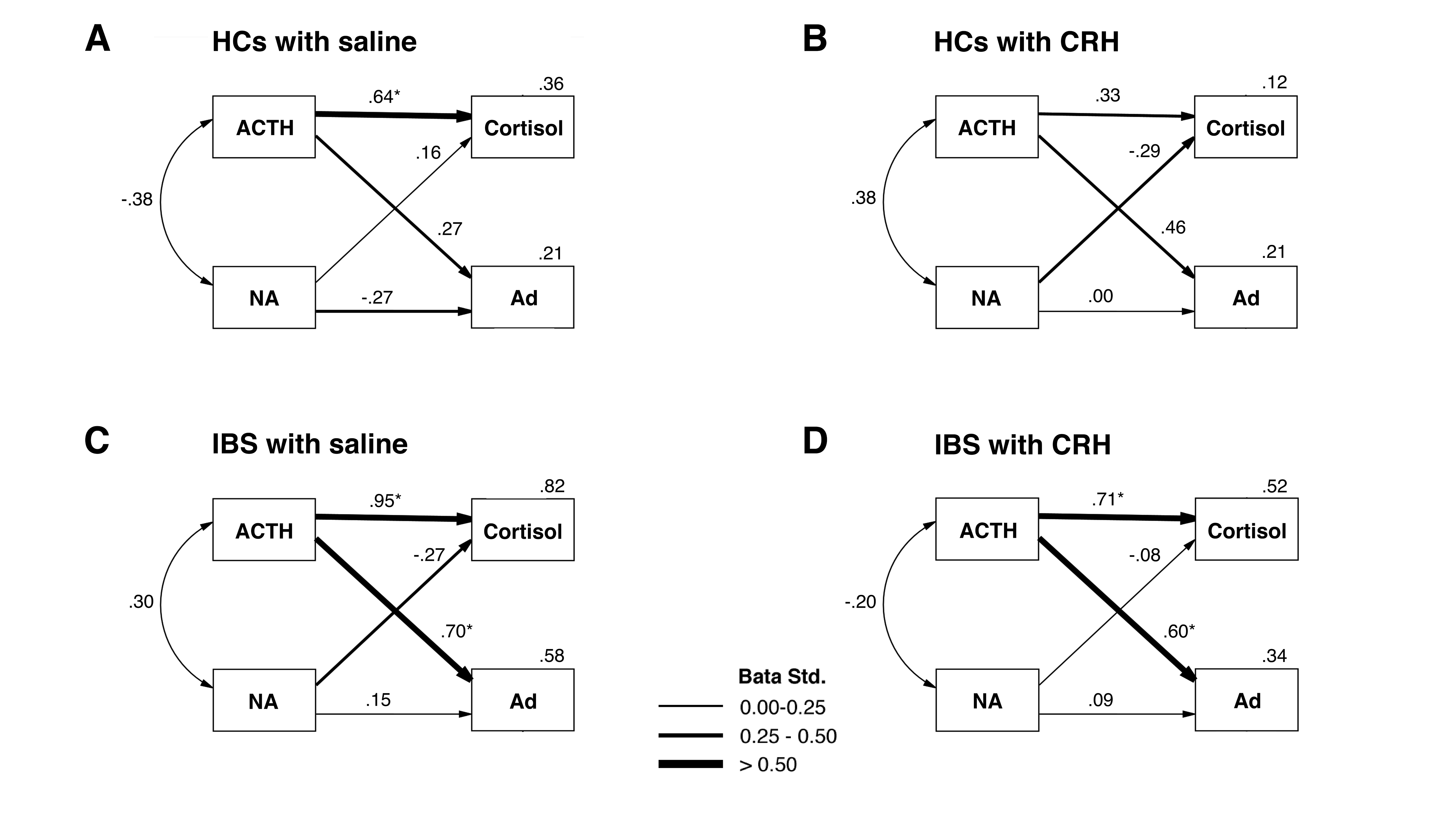

Supplement: S3 Fig — (A) HCs injected with saline (n = 16), (B) HCs injected with CRH (n = 16), (C) patients with IBS injected with saline (n = 16), and (D) patients with IBS injected with CRH (n = 16). *P < .0125 indicate significant paths. The squared multiple correlations (R2) of the variables are reported in the top right corner. There were no significant factor correlations between ACTH and NA. ACTH, plasma ACTH; cortisol, serum cortisol; NA, plasma noradrenaline; Ad, plasma adrenaline; ACTH, adrenocorticotropic hormone; IBS, irritable bowel syndrome; CRH, corticotropin-releasing hormone; HCs, healthy controls. (TIF) [file pone.0199698.s003.tif]

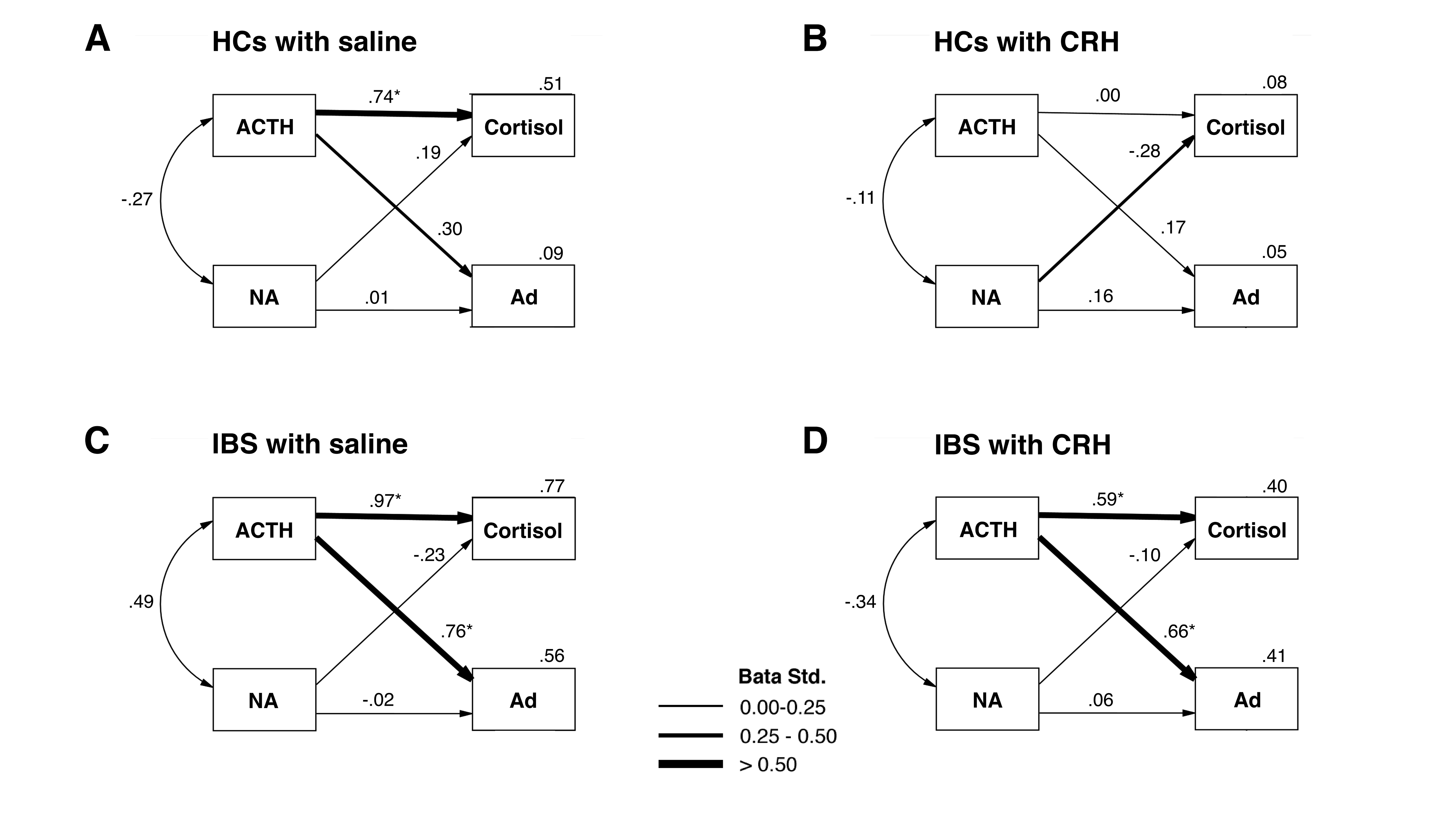

Supplement: S4 Fig — (A) HCs injected with saline (n = 16), (B) HCs injected with CRH (n = 16), (C) patients with IBS injected with saline (n = 16), and (D) patients with IBS injected with CRH (n = 16). *P < .0125 indicate significant paths. The squared multiple correlations (R2) of the variables are reported in the top right corner. There were no significant factor correlations between ACTH and NA. ACTH, plasma ACTH; cortisol, serum cortisol; NA, plasma noradrenaline; Ad, plasma adrenaline; ACTH, adrenocorticotropic hormone; IBS, irritable bowel syndrome; CRH, corticotropin-releasing hormone; HCs, healthy controls. (TIF) [file pone.0199698.s004.tif]
