# Supplementary material for: Relationship between sympathoadrenal and pituitary-adrenal response during colorectal distention in the presence of corticotropin-releasing hormone in patients with irritable bowel syndrome and healthy controls
Source: PLoS One. 2018 Jul 6;13(7):e0199698. doi: 10.1371/journal.pone.0199698 (PMC6034822; doi:10.1371/journal.pone.0199698)
Supplement: S1 Table — (DOCX) [file pone.0199698.s005.docx]

A. GEE result in Female subjects

|  | ACTH (n = 32) | |  | Cortisol (n = 32) | |  | NA (n = 32) | |  | Ad (n = 32) | |
| --- | --- | --- | --- | --- | --- | --- | --- | --- | --- | --- | --- |
|  | Wald χ^2^ | *P* |  | Wald χ^2^ | *P* |  | Wald χ^2^ | *P* |  | Wald χ^2^ | *P* |
| Distention | 5.52 | 0.06 |  | 5.47 | 0.07 |  | 23.68 | < 0.01 |  | 23.08 | < 0.01 |
| Group | 2.30 | 0.13 |  | 0.02 | 0.90 |  | 2.47 | 0.12 |  | 0.18 | 0.67 |
| Drug | 38.82 | < 0.01 |  | 12.13 | < 0.01 |  | 0.02 | 0.89 |  | 0.44 | 0.51 |
| Distention * Group * Drug | 8.75 | 0.01 |  | 1.40 | 0.50 |  | 5.60 | 0.06 |  | 1.49 | 0.48 |

B. GEE result in female and male subjects

|  | ACTH (n = 64) | |  | Cortisol (n = 64) | |  | NA (n = 64) | |  | Ad (n = 64) | |
| --- | --- | --- | --- | --- | --- | --- | --- | --- | --- | --- | --- |
|  | Wald χ^2^ | *P* |  | Wald χ^2^ | *P* |  | Wald χ^2^ | *P* |  | Wald χ^2^ | *P* |
| Distention | 8.27 | 0.02 |  | 5.92 | 0.05 |  | 28.02 | < 0.01 |  | 60.41 | < 0.01 |
| Group | 1.07 | 0.30 |  | 0.08 | 0.77 |  | 4.86 | 0.03 |  | 0.20 | 0.66 |
| Drug | 63.77 | < 0.01 |  | 29.16 | < 0.01 |  | 0.01 | 0.92 |  | 1.42 | 0.23 |
| Sex | 0.12 | 0.73 |  | 0.02 | 0.90 |  | 0.63 | 0.43 |  | 3.07 | 0.08 |
| Distention * Group * Drug | 7.91 | 0.02 |  | 8.54 | 0.01 |  | 17.87 | < 0.01 |  | 11.27 | < 0.01 |
| Distention * Group * Drug * Sex | 5.87 | 0.05 |  | 1.67 | 0.44 |  | 7.27 | 0.03 |  | 2.72 | 0.26 |

Data are shown as Wald χ2 scores of the GEE analysis. Data were used to assess the interactions with colorectal distention, group (IBS, HC), drug (CRH, saline), distention (no distention, 20, and 40 mmHg distention), and sex (female, male) in plasma ACTH, serum cortisol, plasma adrenaline, and noradrenaline levels. ACTH, adrenocorticotropic hormone; Ad, adrenaline; NA, noradrenaline.
