# Supplementary material for: Relationship between sympathoadrenal and pituitary-adrenal response during colorectal distention in the presence of corticotropin-releasing hormone in patients with irritable bowel syndrome and healthy controls
Source: PLoS One. 2018 Jul 6;13(7):e0199698. doi: 10.1371/journal.pone.0199698 (PMC6034822; doi:10.1371/journal.pone.0199698)
Supplement: S2 Table — (DOCX) [file pone.0199698.s006.docx]

A. During no distention

| Variable |  | Mean | SD | ACTH |  | Cortisol |  | Ad |  |
| --- | --- | --- | --- | --- | --- | --- | --- | --- | --- |
| HC with placebo injection (n = 16) | | |  |  |  |  |  |  |  |
|  | ACTH | 28.35 | 13.43 | - |  |  |  |  |  |
|  | Cort | 13.56 | 3.32 | 0.58 | * | - |  |  |  |
|  | Ad | 16.75 | 11.55 | 0.37 |  | 0.11 |  | - |  |
|  | NA | 143.38 | 101.85 | -0.34 |  | 0.10 |  | -0.36 |  |
| HC with CRH injection (n = 16) | | |  |  |  |  |  |  |  |
|  | ACTH | 80.77 | 43.94 | - |  |  |  |  |  |
|  | Cort | 17.26 | 4.17 | 0.23 |  | - |  |  |  |
|  | Ad | 16.69 | 10.16 | 0.58 | * | 0.48 |  | - |  |
|  | NA | 139.25 | 52.15 | 0.33 |  | -0.26 |  | 0.09 |  |
| IBS with placebo injection (n = 16) | | |  |  |  |  |  |  |  |
|  | ACTH | 28.98 | 21.44 | - |  |  |  |  |  |
|  | Cort | 12.87 | 5.54 | 0.62 | ** | - |  |  |  |
|  | Ad | 22.56 | 21.73 | 0.41 |  | 0.05 |  | - |  |
|  | NA | 171.06 | 107.69 | 0.44 |  | 0.03 |  | 0.49 |  |
| IBS with CRH injection (n = 16) | | |  |  |  |  |  |  |  |
|  | ACTH | 71.18 | 28.13 | - |  |  |  |  |  |
|  | Cort | 16.74 | 3.14 | 0.59 | * | - |  |  |  |
|  | Ad | 16.31 | 7.94 | 0.53 | * | 0.23 |  | - |  |
|  | NA | 194.38 | 92.13 | -0.10 |  | -0.15 |  | 0.09 |  |

B. During 20 mmHg distention

| Variable |  | Mean | SD | ACTH |  | Cortisol |  | Ad |  |
| --- | --- | --- | --- | --- | --- | --- | --- | --- | --- |
| HC with placebo injection (n = 16) | | |  |  |  |  |  |  |  |
|  | ACTH | 28.09 | 13.56 | - |  |  |  |  |  |
|  | Cort | 13.05 | 3.76 | 0.55 | * | - |  |  |  |
|  | Ad | 17.56 | 13.70 | 0.36 |  | 0.23 |  | - |  |
|  | NA | 148.06 | 99.55 | -0.42 |  | -0.04 |  | -0.27 |  |
| HC with CRH injection (n = 16) | | |  |  |  |  |  |  |  |
|  | ACTH | 76.03 | 25.75 | - |  |  |  |  |  |
|  | Cort | 17.92 | 4.11 | 0.00 |  | - |  |  |  |
|  | Ad | 26.88 | 24.23 | 0.07 |  | 0.16 |  | - |  |
|  | NA | 146.56 | 73.06 | -0.22 |  | -0.22 |  | 0.16 |  |
| IBS with placebo injection (n = 16) | | |  |  |  |  |  |  |  |
|  | ACTH | 28.40 | 17.92 | - |  |  |  |  |  |
|  | Cort | 13.28 | 4.52 | 0.72 | ** | - |  |  |  |
|  | Ad | 26.50 | 22.18 | 0.28 |  | 0.42 |  | - |  |
|  | NA | 185.69 | 108.79 | 0.56 | * | 0.28 |  | 0.16 |  |
| IBS with CRH injection (n = 16) | | |  |  |  |  |  |  |  |
|  | ACTH | 72.77 | 25.43 | - |  |  |  |  |  |
|  | Cort | 17.64 | 3.38 | 0.59 | * | - |  |  |  |
|  | Ad | 21.88 | 11.57 | 0.57 | * | 0.67 | ** | - |  |
|  | NA | 192.00 | 85.89 | -0.25 |  | -0.31 |  | -0.06 |  |

C. During 40 mmHg distention

| Variable |  | Mean | SD | ACTH |  | Cortisol |  | Ad |  |
| --- | --- | --- | --- | --- | --- | --- | --- | --- | --- |
| HC with placebo injection (n = 16) | | |  |  |  |  |  |  |  |
|  | ACTH | 27.09 | 14.18 | - |  |  |  |  |  |
|  | Cort | 11.63 | 3.58 | 0.44 |  | - |  |  |  |
|  | Ad | 25.63 | 17.37 | 0.40 |  | 0.06 |  | - |  |
|  | NA | 151.06 | 101.75 | -0.61 | * | 0.08 |  | -0.17 |  |
| HC with CRH injection (n = 16) | | |  |  |  |  |  |  |  |
|  | ACTH | 105.84 | 72.07 | - |  |  |  |  |  |
|  | Cort | 18.63 | 3.70 | 0.19 |  | - |  |  |  |
|  | Ad | 67.69 | 66.53 | 0.30 |  | 0.23 |  | - |  |
|  | NA | 166.31 | 63.39 | 0.30 |  | -0.10 |  | 0.50 |  |
| IBS with placebo injection (n = 16) | | |  |  |  |  |  |  |  |
|  | ACTH | 30.58 | 23.28 | - |  |  |  |  |  |
|  | Cort | 12.66 | 4.66 | 0.82 | ** | - |  |  |  |
|  | Ad | 37.31 | 28.25 | 0.61 | * | 0.49 |  | - |  |
|  | NA | 221.75 | 168.33 | 0.46 |  | 0.34 |  | 0.31 |  |
| IBS with CRH injection (n = 16) | | |  |  |  |  |  |  |  |
|  | ACTH | 74.21 | 35.13 | - |  |  |  |  |  |
|  | Cort | 17.34 | 3.22 | 0.62 | * | - |  |  |  |
|  | Ad | 33.13 | 16.63 | 0.27 |  | 0.26 |  | - |  |
|  | NA | 194.06 | 84.10 | 0.06 |  | 0.19 |  | -0.26 |  |

Data are shown as rho scores of the Spearman rank correlation coefficient. Data were used to assess the correlations with plasma ACTH, serum cortisol, plasma adrenaline, and noradrenaline levels during (A) no distention, (B) 20 mm Hg distention, and (C) 40 mm Hg distention. * *P* < 0.05, ** *P* < 0.01. ACTH, adrenocorticotropic hormone; Ad, adrenaline; NA, noradrenaline; SD, standard deviation.
