# Supplementary material for: Relationship between sympathoadrenal and pituitary-adrenal response during colorectal distention in the presence of corticotropin-releasing hormone in patients with irritable bowel syndrome and healthy controls
Source: PLoS One. 2018 Jul 6;13(7):e0199698. doi: 10.1371/journal.pone.0199698 (PMC6034822; doi:10.1371/journal.pone.0199698)
Supplement: S1 Text — (DOCX) [file pone.0199698.s007.docx]

We compared the effects of CRH administration on HPA responses to colonic distention between the two groups using GEE analysis of neuroendocrine levels with group (IBS or HC) and drug (CRH or placebo) as the between-subject factors, and distention as the within-subject factor in female participants (**S1 Fig**). There was a significant distention × group × drug interaction effect (*P* = .013) for plasma ACTH levels, but not for serum cortisol levels (*P* = .497) (**S1 Table**). We assessed the effects of CRH on adrenergic responses and found no significant distention × group × drug interaction effect for plasma noradrenaline or adrenaline levels (noradrenaline: *P* = .061, adrenaline: *P* = .475) (**S1 Table**). To investigate the effects of sex, we assessed the distention × group × drug × sex interaction using GEE analysis between female and male participants whose hormonal responses have been reported. We found only a significant distention × group × drug × sex interaction for plasma noradrenaline levels (*P* = .026) (**S1 Table**). The basal levels of plasma ACTH (IBS, 19.4 ± 9.4 pg/ml; HC, 18.9 ± 10.8 pg/ml), serum cortisol (IBS, 12.4 ± 3.5 μg/ml; HC, 11.5 ± 4.9 μg/ml), and plasma noradrenaline (IBS, 123.8 ± 67.5 pg/ml; HC, 152.3 ± 75.2 pg/ml) were not significantly different between the female IBS group and the HCs. Basal plasma adrenaline (IBS, 14.2 ± 8.1 pg/ml; HC, 8.8 ± 5.0 pg/ml) levels in female patients with IBS were significantly higher than those in HCs (*P* = .033). To understand the effects of CRH injection, neuroendocrine levels were compared between the CRH injection and placebo conditions. Plasma ACTH levels were significantly higher in both HC and IBS groups receiving a CRH injection when compared with the placebo groups (IBS, *P* = .006; HC, *P* = .003). In addition, plasma noradrenaline levels in the IBS group receiving CRH were significantly higher than those in the IBS group receiving placebo (*P* = .046) **(S2 Fig)**.
