# Supplementary material for: Relationship between sympathoadrenal and pituitary-adrenal response during colorectal distention in the presence of corticotropin-releasing hormone in patients with irritable bowel syndrome and healthy controls
Source: PLoS One. 2018 Jul 6;13(7):e0199698. doi: 10.1371/journal.pone.0199698 (PMC6034822; doi:10.1371/journal.pone.0199698)
Supplement: S2 Text — (DOCX) [file pone.0199698.s008.docx]

To assess the effects of CRH injection, subjective symptoms in female participants were compared before and after CRH injection. We found that the IBS group (placebo) had higher stress and sleep levels than the HC group (placebo) during baseline (stress, *P* = .037; sleep, *P* = .004). Moreover, during random distention after drug injection, we found significant distention × group × drug interactions for stress and anxiety levels (stress, *P* = .016; anxiety, *P* = .014).
